# Investigation of gene–diet interactions in the incretin system and risk of type 2 diabetes: the EPIC-InterAct study

**DOI:** 10.1007/s00125-016-4090-5

**Published:** 2016-09-13

**Authors:** The InterAct Consortium

**Affiliations:** c/o A. Heraclides, University of Nicosia Medical School, Centre for Primary Care and Population Health, 21 Ilia Papakyriakou, Engomi, P.O. Box 24005, 1700 Nicosia Cyprus

**Keywords:** Coffee, Dairy, Fibre, Gene–environment interaction, *GIPR*, Incretins, *KCNQ1*, Olive oil, *TCF7L2*, *WFS1*

## Abstract

**Aims/hypothesis:**

The gut incretin hormones glucagon-like peptide-1 (GLP-1) and glucose-dependent insulinotropic peptide (GIP) have a major role in the pathophysiology of type 2 diabetes. Specific genetic and dietary factors have been found to influence the release and action of incretins. We examined the effect of interactions between seven incretin-related genetic variants in *GIPR*, *KCNQ1*, *TCF7L2* and *WFS1* and dietary components (whey-containing dairy, cereal fibre, coffee and olive oil) on the risk of type 2 diabetes in the European Prospective Investigation into Cancer and Nutrition (EPIC)-InterAct study.

**Methods:**

The current case-cohort study included 8086 incident type 2 diabetes cases and a representative subcohort of 11,035 participants (median follow-up: 12.5 years). Prentice-weighted Cox proportional hazard regression models were used to investigate the associations and interactions between the dietary factors and genes in relation to the risk of type 2 diabetes.

**Results:**

An interaction (*p* = 0.048) between *TCF7L2* variants and coffee intake was apparent, with an inverse association between coffee and type 2 diabetes present among carriers of the diabetes risk allele (T) in rs12255372 (GG: HR 0.99 [95% CI 0.97, 1.02] per cup of coffee; GT: HR 0.96 [95% CI 0.93, 0.98]); and TT: HR 0.93 [95% CI 0.88, 0.98]). In addition, an interaction (*p* = 0.005) between an incretin-specific genetic risk score and coffee was observed, again with a stronger inverse association with coffee in carriers with more risk alleles (0–3 risk alleles: HR 0.99 [95% CI 0.94, 1.04]; 7–10 risk alleles: HR 0.95 [95% CI 0.90, 0.99]). None of these associations were statistically significant after correction for multiple testing.

**Conclusions/interpretation:**

Our large-scale case-cohort study provides some evidence for a possible interaction of *TCF7L2* variants and an incretin-specific genetic risk score with coffee consumption in relation to the risk of type 2 diabetes. Further large-scale studies and/or meta-analyses are needed to confirm these interactions in other populations.

**Electronic supplementary material:**

The online version of this article (doi:10.1007/s00125-016-4090-5) contains peer-reviewed but unedited supplementary material, which is available to authorised users.

## Introduction

The gut incretin hormones glucagon-like peptide-1 (GLP-1) and glucose-dependent insulinotropic peptide (GIP) have a major role in acute food-stimulated secretion of insulin but also affect long-term beta cell mass and functioning [1]. Single nucleotide polymorphism (SNPs) in genes associated with type 2 diabetes in genome-wide association studies (GWAS) have been found to regulate the release and functioning of incretin hormones in small-scale experimental studies [2, 3]. Genetic variants in transcription factor 7-like 2 (*TCF7L2*) [4–8] and Wolframin ER transmembrane glycoprotein (*WFS1*) [9] have been linked to pancreatic GLP-1 sensitivity, gastric inhibitory polypeptide receptor (*GIPR*) variants have been linked to pancreatic GIP sensitivity [2], and genetic variants in potassium voltage-gated channel subfamily Q member 1 (*KCNQ1*) have been reported to affect GLP-1 and GIP secretion from the gut [10–12].

Despite the direct involvement of food ingestion in the incretin response, the role of diet in the regulation of the incretin system is largely unknown. Some dietary factors have been found to stimulate the release and action of incretin hormones in animal studies and small-scale experimental studies in humans. Whey protein (present in all dairy products except cheese) has been found to increase postprandial GIP and GLP-1 concentrations compared with isoenergetic non-whey containing meals [13–15] or dairy casein protein [16, 17]. Furthermore, whey-containing dairy products have been found to have a disproportionally high insulin index (typical blood insulin response to various foods) compared with their corresponding glycaemic index [18]. Similarly, intake of olive oil has been found to increase the production of both GIP and GLP-1 in healthy individuals [19] and GLP-1 in individuals with type 2 diabetes [20] compared with an isoenergetic intake of butter. A postprandial increase in the level of intact GLP-1 has also been found after consumption of purified oleic acid in animal studies [21]. Dietary fibre, especially of cereal origin, has also been found to stimulate GIP and GLP-1 release in healthy individuals [22, 23]. Finally, coffee, via mechanisms involving chlorogenic acid (the major polyphenol in coffee), has been found to increase production of GLP-1 compared with isoenergetic beverages [24].

All of the above dietary factors have also been linked to a reduction in the risk of type 2 diabetes in epidemiological studies [25]. In the European Prospective Investigation into Cancer and Nutrition (EPIC) - InterAct study, an inverse association between dairy products and type 2 diabetes was found [26], while dietary fibre and olive oil did not show an association [27, 28].

We hypothesised that the association between specific foods potentially influencing incretin release (whey-containing dairy, cereal fibre, olive oil and coffee) and type 2 diabetes risk is modified by incretin-related genetic variants. We investigated this hypothesis in the large population-based EPIC-InterAct study conducted in eight European countries.

## Methods

### Study population

The design and methods of the InterAct study, nested within the EPIC cohorts, are described in detail elsewhere [29]. All participants provided written informed consent, and the study was approved by the local ethics committee in the participating countries and the internal review board of the International Agency for Research on Cancer. Briefly, the study population included participants from 26 centres in the eight of the ten countries participating in EPIC who had available blood samples and information on diabetes (France, Italy, Spain, the UK, the Netherlands, Germany, Denmark and Sweden). All ascertained and verified incident type 2 diabetes cases between 1991 and 2007 (3.99 million person-years at risk, *n* = 12,403) were included in the case group. A centre-stratified, representative subcohort of 16,835 individuals was selected as the control group. Prevalent diabetes cases (*n* = 548) and individuals with uncertain diabetes status (*n* = 133) were excluded from the subcohort, leaving 16,154 individuals for analysis. Due to the random selection of the control group, 778 incident type 2 diabetes cases were part of the subcohort, leaving a total study sample of 27,779 individuals.

For the current analysis, we excluded participants with abnormal estimated energy intake (top 1% and bottom 1% of the distribution of the ratio of reported energy intake over estimated energy requirements, assessed by the basal metabolic rate) (*n* = 619) and those with missing information on dietary intake (*n* = 117). Participants with no data on any of the SNPs of interest (including all Danish participants) (*n* = 7617) were also excluded. For our interaction analysis, the models were adjusted for demographic and lifestyle factors, thus participants with no information on these key covariates were also excluded. Therefore, 18,638 individuals were included in the analysis, comprising of 8086 cases and 11,035 subcohort participants (including 483 cases in the subcohort). For some of these participants, information on specific SNPs was missing, thus the sample size for the analysis of each SNP differs slightly.

### Case ascertainment

Ascertaining incident type 2 diabetes involved a review of the existing EPIC datasets at each centre using multiple sources of evidence including self-report, linkage to disease and drug registers, hospital admissions and mortality data. Information from any follow-up visit or external evidence with a date later than the baseline visit was used. To increase the specificity of the definition for these cases, we sought further evidence including individual medical records review in some centres. Follow-up was censored at the date of diagnosis, 31 December 2007 or the date of death, whichever occurred first. In total, 12,403 verified incident type 2 diabetes cases were identified.

### Choice of dietary factors and dietary assessment

After a detailed literature review, we identified four dietary factors (whey-containing dairy, olive oil, coffee and cereal fibre) for which there was evidence for increasing postprandial incretin levels [13–15]. Self- or interviewer-administered country-specific validated dietary questionnaires and/or diet records (Sweden only) were used to assess the usual food intake of participants. Nutrient intake was estimated using the standardised EPIC nutrient database (ENDB) [30]. Whey-containing dairy was calculated by subtracting the intake of cheese from the total intake of dairy products including milk, yoghurt, milk-based puddings and cream desserts. Olive oil consumption was reported on its own or as an ingredient in recipes, depending on the country. In the Swedish study centre Umeå, olive oil was not assessed in the questionnaire, and in the UK, it was only reported as an ingredient in recipes. Therefore, these centres had to be excluded from the olive oil interaction analysis. Coffee intake included both caffeinated and decaffeinated coffee. Cereal fibre was derived from the ENDB by adding the fibre content of cereal-based products, including bread, rice, wheat-based pasta, crisp bread, rusks and breakfast cereals [31].

### Covariate assessment

Questionnaires were used to collect information on lifestyle factors and socioeconomic status at baseline [32]. For the current analysis we used a four category physical activity index reflecting occupational and recreational physical activity [33]. Educational attainment was categorised as: none; primary school; technical school; secondary school; and further education including university degree. Smoking status was categorised as: never; former; and current smoker. Alcohol consumption was categorised as: 0 g/day; >0–6 g/day; >6–12 g/day; >12–24 g/day; and >24 g/day. Total energy intake was assessed as kcal/day (converted to MJ/day), and intake of specific foods and nutrients of interest as g/day. Anthropometric measures including weight, height and waist circumference were collected at baseline by standardised procedures and adjusted for clothing [34]. Information on prevalent diseases was obtained at baseline including stroke, myocardial infarction, hypertension and hyperlipidaemia.

### DNA extraction, genotyping and SNP selection

DNA was extracted from up to 1 ml of buffy coat for each individual from a citrated blood sample. A detailed account on the DNA extraction and genotyping procedures has been published previously [35]. Briefly, a total of 10,027 participants (4644 cases) were randomly selected across all centres (except Denmark) for genome-wide genotyping using the Illumina 660 W-Quad BeadChip (Illumina, San Diego, CA, USA). In addition, 9794 EPIC-InterAct participants with available DNA and not selected for genome-wide measurement were genotyped using the Illumina Cardio-Metabochip (Illumina, San Diego, CA, USA) [35].

Seven SNPs (*GIPR*: rs10423928; *TCF7L2*: rs7903146 and rs12255372; *WFS1*: rs10010131; *KCNQ1*: rs151290, rs2237892 and rs163184) associated with type 2 diabetes in GWAS [36] were selected based on evidence from small-scale experimental studies that had revealed major roles in the regulation of the release and functioning of incretin hormones [2, 3, 7, 8]. As *TCF7L2* has several point mutations implicated in type 2 diabetes, we chose to use the ones for which there is evidence for involvement in the incretin system, based on small-scale experimental studies in humans. More specifically, two independent studies have collectively shown that *TCF7L2* rs7903146, rs7901695 and rs12255372 influence the second phase of GLP-1-induced insulin secretion [7, 8]. Since *TCF7L2* rs7903146 and *TCF7L2* rs7901695 are in strong linkage disequilibrium (LD) (R^2^ = 0.98, D’ = 0.88) it is likely that the two SNPs capture the same genetic information, thus of the two, we only included rs7903146 in our analysis. The *KCNQ1* SNPs included in our analysis were in low LD (R^2^ < 0.25).

Four of the aforementioned seven SNPs (*GIPR* rs10423928, *TCF7L2* rs7903146, *WFS1* rs10010131 and *KCNQ1* rs163184) were available from direct genotyping within the entire EPIC-InterAct study on Sequenom or Taqman platforms. Information on two of the remaining SNPs (*TCF7L2* rs12255372 and *KCNQ1* rs2237892) was available from genotyping with the GWAS Illumina 660 W-Quad Chip and Illumina Cardio-Metabochip. The SNP *KCNQ1* rs151290 was not available on any of the genotyping arrays and *KCNQ1* rs163171 was chosen as a proxy SNP (r^2^ = 0.94, D’ = 1.0 in the CEU [Utah Residents with Northern and Western European Ancestry] of 1000 Genomes phase 3 dataset assessed via www.ensembl.org, accessed 21 January 2015). No significant deviation from Hardy–Weinberg equilibrium was observed (*p* > 0.01).

An unweighted genetic risk score was constructed based on all unlinked genetic variants (*TCF7L2* rs7903146, *KCNQ1* rs163184, *KCNQ1* rs2237892, *GIPR* rs10423928 and *WFS1* rs10010131) that were significantly related to type 2 diabetes within the study population. Minor allele frequencies for all SNPs can be found in the electronic supplementary material (ESM) Table 1.

### Statistical analyses

Prentice-weighted Cox regression was used with age as the underlying time-scale, incident type 2 diabetes as the outcome and each SNP (*GIPR*: rs10423928; *TCF7L2*: rs7903146 and rs12255372; *WFS1*: rs10010131; *KCNQ1*: rs151290, rs2237892 and rs163184) as well as the genetic risk score, in turn, as exposure variables, stratified by centre and age at baseline (rounded to the nearest integer) and adjusted for sex. For each SNP, per genotype and additive genetic effects were modelled using the minor allele as the effect allele. Gene–diet interactions for each SNP (as well as genetic risk score) and intake of each dietary factor (whey-containing dairy, cereal fibre, coffee and olive oil) were modelled assuming an additive genetic effect by inclusion of a multiplicative interaction term in the model. This model was further adjusted for physical activity, education, BMI, smoking status, total energy intake, intake of fruit and vegetables, meat, soft drinks and alcohol, and mutual adjustment of the four dietary factors of interest. All dietary factors, except coffee, were energy-adjusted by the residual method [37]. Dietary factors were scaled to represent one serving. Calculation of one serving for each food item was based on previous publications from the EPIC-InterAct study and main EPIC studies: dairy 150 g/day; cereal fibre 10 g/day; olive oil 10 g/day; and coffee 125 g/day [26, 38, 39].

The country-specific Cox regression coefficients in the genetic main effects analysis and gene–diet interaction analyses were estimated and combined using random-effects meta-analysis. Between-study heterogeneity was estimated using I^2^.

In the interaction analysis, we established the number of independent genetic variables from a correlation matrix (Pearson’s r) of all genetic variables tested in the interaction analysis (seven SNPs), and a genetic risk score (range 0–10) by spectral decomposition [40]. Based on the number of independent genetic variables (*n* = 7) and dietary factors (*n* = 4) tested, *p* values below the multiple testing corrected significance threshold of 0.0018 (=0.05/28) were considered to be statistically significant.

All statistical analyses were performed using the SAS Enterprise Guide 6.1 (SAS Institute, Cary, NC, USA) and SAS 9.4 (SAS Institute). Meta-analyses were performed using R (version 3.1.2, www.r-project.org) and the R function ‘metagen’ available from the R package ‘meta’ (version 4.3-0) in PROC IML.

## Results

Participants in the subcohort (*n* = 11,035) were followed up for a median (interquartile range, IQR) of 12.5 (2.4) years, and 64% were women. The population was on average middle-aged (median [IQR] age 51.5 [14.0]) and had a median (IQR) BMI of 25.5 (5.4) kg/m^2^ (Table 1). The median (IQR) intake of the dietary factors of interest was 7.2 (5.2) g/day for cereal fibre, 248 (280) g/day for whey-containing dairy products, 1.7 (19.9) g/day for olive oil and 233 (417) g/day for coffee (Table 1). Study population characteristics stratified by country can be found in ESM Table 2.

**Table 1:** Baseline characteristics of the EPIC-InterAct subcohort participants with data available for at least one SNP of interest

Characteristic	Participants with missing data (%)	Subcohort participants (*n* = 11,035)
Age (years)	0	51.5 (45.0–59.0)
Sex (female)	0	7065 (64.0)
BMI (kg/m^2^)	0	25.5 (23.0–28.4)
Waist (cm)	8.9	85.0 (76.0–94.5)
Highest school level	0	
None		943 (8.6)
Primary		3613 (32.7)
Technical		2418 (21.9)
Secondary		1745 (15.8)
Further education		2316 (21.0)
Smoking status	0	
Never		5356 (48.5)
Former		2941 (26.7)
Current		2738 (24.8)
Cambridge Index of Physical Activity	0	
Inactive		2754 (25.0)
Moderately inactive		3800 (34.4)
Moderately active		2500 (22.7)
Active		1981 (18.0)
Energy intake (MJ/day)	0	8.5 (6.9–10.5)
Cereal fibre (g/day)	0	7.2 (5.0–10.2)
Fruit and vegetables (g/day)	0	310 (189–467)
Whey-containing dairy products (g/day)	0	248 (129–409)
Meat (g/day)	0	70.0 (43.9–103.4)
Olive oil (g/day)	17.0	1.7 (0.0–19.9)
Coffee (g/day)	0	233 (83–500)
Soft drinks (g/day)	0	2.6 (0.0–64.3)
Alcohol intake (g/day)	0	
0		1928 (17.5)
>0–6		3976 (36.0)
>6–12		1649 (14.9)
>12–24		1709 (15.5)
>24		1773 (16.1)
Hypertension	0.6	2116 (19.2)
Hyperlipidaemia	24.8	1617 (14.7)
Stroke	10.4	78 (0.7)
Myocardial infarction	1.8	134 (1.2)

Data are median (25th–75th percentile) or number (%)

All selected SNPs, with the exception of *KCNQ1* rs163171 (the only proxy SNP used in our analysis), were associated with incident type 2 diabetes (ESM Table 3). We found no evidence of an interaction with any of the dietary factors for *WFS1* rs10010131, or *KCNQ1* rs163171, rs163184 and rs2237892. For *GIPR* rs10423928, we observed a marginally non-significant interaction with olive oil (*p* = 0.050) (Table 2).

**Table 2:** Multivariable-adjusted HR (95% CI) for risk of type 2 diabetes per one serving per day increment of each dietary factor, stratified by incretin-related SNPs, in the EPIC-InterAct study (*n* = 18,638)

SNP	HR (95% CI)			*p*_Interaction_
*TCF7L2* rs12255372 (*n*_cases_ */n*_total_)	GG (3,216/8,171)	GT (3,520/7,661)	TT^a^ (906/1,807)	
Whey-containing dairy (150 g/day)	1.04 (1.00, 1.08)	0.98 (0.94, 1.02)	1.06 (0.97, 1.15)	0.67
Cereal fibre (10 g/day)	0.94 (0.81, 1.09)	1.06 (0.92, 1.23)	1.35 (0.97, 1.88)	0.08
Coffee (125 g/day)	0.99 (0.97, 1.02)	0.96 (0.93, 0.98)	0.93 (0.88, 0.98)	0.048
Olive oil (10 g/day) (*n*_cases_ */n*_total_)^b^	0.97 (0.91, 1.04) (2,546/6,459)	0.98 (0.92, 1.04) (2,919/6,475)	0.97 (0.88, 1.08) (781/1,585)	0.67
*TCF7L2* rs7903146 (*n*_cases_ */n*_total_)	CC (3,197/8,196)	CT (3,622/7,896)	TT^a^ (977/1,879)	
Whey-containing dairy (150 g/day)	1.04 (1.00, 1.08)	0.99 (0.95, 1.03)	1.02 (0.94, 1.11)	0.89
Cereal fibre (10 g/day)	0.94 (0.81, 1.09)	1.06 (0.92, 1.24)	1.13 (0.83, 1.54)	0.30
Coffee (125 g/day)	0.99 (0.97, 1.01)	0.95 (0.93, 0.98)	0.95 (0.90, 1.00)	0.08
Olive oil (10 g/day) (*n*_cases_ */n*_total_)^b^	0.98 (0.92, 1.04) (2,539/6,500)	1.00 (0.94, 1.06) (3,002/6,659)	0.90 (0.81, 0.98) (835/1,630)	0.90
*KCNQ1* rs163171 (*n*_cases_ */n*_total_)	CC (4,647/10,652)	CT (2,605/6,110)	TT^a^ (391/883)	
Whey-containing dairy (150 g/day)	1.03 (0.99, 1.06)	1.01 (0.96, 1.05)	1.09 (0.94, 1.25)	0.77
Cereal fibre (10 g/day)	1.04 (0.92, 1.19)	0.93 (0.79, 1.11)	0.64 (0.36, 1.16)	0.18
Coffee (125 g/day)	0.98 (0.96, 1.00)	0.95 (0.92, 0.98)	1.03 (0.95, 1.11)	0.75
Olive oil (10 g/day) (*n*_cases_ */n*_total_)^b^	0.99 (0.94, 1.04) (3,862/8,841)	0.95 (0.89, 1.02) (2,083/4,985)	0.97 (0.78, 1.22) (302/699)	0.56
*KCNQ1* rs163184 (*n*_cases_ */n*_total_)	TT (2,007/4,850)	GT (3,971/9,135)	GG^a^ (1,977/4,367)	
Whey-containing dairy (150 g/day)	1.01 (0.96, 1.07)	1.00 (0.97, 1.04)	1.02 (0.97, 1.07)	0.59
Cereal fibre (10 g/day)	0.97 (0.80, 1.17)	0.89 (0.77, 1.03)	1.17 (0.98, 1.40)	0.69
Coffee (125 g/day)	0.97 (0.94, 1.00)	0.97 (0.95, 0.99)	0.97 (0.93, 1.00)	0.22
Olive oil (10 g/day) (*n*_cases_ */n*_total_)^b^	1.00 (0.93, 1.08) (1,668/4,025)	0.96 (0.91, 1.01) (3,240/7,551)	0.94 (0.87, 1.01) (1,591/3,529)	0.97
*KCNQ1* rs2237892 (*n*_cases_ */n*_total_)	CC^a^ (6,148/13,869)	CT (701/1,647)	TT (20/61)	
Whey-containing dairy (150 g/day)	1.01 (0.98, 1.04)	1.09 (1.00, 1.19)	–^c^	0.56
Cereal fibre (10 g/day)	0.99 (0.88, 1.11)	0.97 (0.69, 1.37)	–^c^	0.81
Coffee (125 g/day)	0.98 (0.96, 0.99)	0.97 (0.92, 1.02)	–^c^	0.27
Olive oil (10 g/day) (*n*_cases_ */n*_total_)^b^	0.95 (0.91, 0.99) (5,100/11,579)	1.17 (0.98, 1.39) (529/1,250)	–^c^ (13/38)	0.07
*GIPR* rs10423928 (*n*_cases_/*n*_total_)	TT (4,925/11,548)	AT (2,684/6,014)	AA^a^ (345/784)	
Whey-containing dairy (150 g/day)	1.04 (1.00,1.07)	0.99 (0.95, 1.04)	1.04 (0.90, 1.22)	0.51
Cereal fibre (10 g/day)	0.98 (0.87, 1.12)	1.18 (1.00, 1.40)	0.66 (0.43, 1.01)	0.55
Coffee (125 g/day)	0.97 (0.95, 0.99)	0.97 (0.94, 0.99)	0.93 (0.85, 1.02)	0.92
Olive oil (10 g/day) (*n*_cases_ */n*_total_)^b^	0.93 (0.88, 0.98) (3,989/9,439)	1.04 (0.97, 1.11) (2,226/5,003)	1.38 (1.02, 1.85) (283/657)	0.050
*WFS1* rs10010131 (*n*_cases_/*n*_total_)	GG^a^ (2,857/6,392)	AG (3,424/8,095)	AA (1,185/2,773)	
Whey-containing dairy (150 g/day)	1.00 (0.96, 1.04)	1.04 (1.00, 1.08)	0.99 (0.92, 1.07)	0.95
Cereal fibre (10 g/day)	1.02 (0.86, 1.20)	0.99 (0.85, 1.14)	0.81 (0.63, 1.04)	0.72
Coffee (125 g/day)	0.97 (0.95, 1.00)	0.98 (0.96, 1.00)	0.94 (0.91, 0.98)	0.49
Olive oil (10 g/day) (*n*_cases_ */n*_total_)^b^	0.98 (0.92, 1.04) (2,378/5,346)	0.97 (0.91, 1.03) (2,783/6,616)	0.92 (0.84, 1.01) (935/2,215)	0.27

Cox regression models were stratified by centre and age (years) and adjusted for sex, study centre, educational attainment, physical activity, smoking status, BMI, alcohol consumption, soft drink consumption, fruit and vegetable intake, meat intake, total energy intake, and mutual adjustment of the four dietary factors of interest (whey-containing-dairy, cereal fibre, olive oil and coffee)

^a^Type 2 diabetes risk allele

^b^Participants in the UK centres (*n*_cases_ = 685/*n*_total_ = 1594) and the Swedish centre Umeå (*n*_cases_ = 785/*n*_total_ = 1693) were excluded from this specific analysis due to inaccurate assessment of olive oil consumption

^c^Numbers are too low to estimate HR for rare allele homozygotes

We identified a nominally significant interaction between a *TCF7L2* variant and coffee consumption (rs12255372: *p*_Interaction_ = 0.048, Table 2). Overall, coffee intake was inversely related to the risk of type 2 diabetes (ESM Table 4). Stratification by rs12255372 genotype indicated a lack of an association between coffee intake and type 2 diabetes in non-carriers of the risk allele (rs12255372-GG: HR 0.99 [95% CI 0.97, 1.02] per cup of coffee; Table 2), while with each additional type 2 diabetes risk allele, the inverse association between coffee intake and risk of type 2 diabetes became stronger (rs12255372-GT: HR 0.96 [95% CI 0.93, 0.98] per cup; rs12255372-TT: HR 0.93 [95% CI 0.88, 0.98] per cup; Table 2). *TCF7L2* rs12255372 is in moderate LD to *TCF7L2* rs7903146 (see Methods section) for which we observed a similar trend for interaction with coffee (Table 2). There was no evidence for heterogeneity between countries (I^2^ = 8.8%).

We also observed some effect modification in the association between cereal fibre and type 2 diabetes by *TCF7L2* variants, but the interaction terms did not reach statistical significance. As observed for coffee, the interaction of cereal fibre with *TCF7L2* SNPs was slightly stronger for rs12255372 (*p*_Interaction_ = 0.08, Table 2) than for rs7903146 (*p*_Interaction_ = 0.30, Table 2).

A genetic risk score based on the diabetes risk alleles *TCF7L2* rs7903146, *KCNQ1* rs163184, *KCNQ1* rs2237892, *GIPR* rs10423928 and *WFS1* rs10010131 also showed a nominally significant interaction with coffee intake (*p* = 0.005, Table 3). The inverse association between coffee intake and risk of type 2 diabetes was stronger in carriers of six or more incretin-specific risk alleles (Table 3). After exclusion of *TCF7L2* rs7903146 from the risk score the interaction effect remained significant, indicating that the effect was not only driven by the *TCF7L2* SNP that showed a trend for interaction with coffee in the single SNP analysis. None of the abovementioned interactions can be considered significant after correction for multiple testing.

**Table 3:** Multivariable-adjusted HR (95% CI) stratified by genetic risk score and *p* value for the interaction of each dietary factor with incident type 2 diabetes in the EPIC-InterAct study (*n* = 18,638)

	Genetic risk score^a^					*p*_Interaction_
	0–3	4	5	6	7–10	
*n*_cases_/*n*_total_	642/1710	1188/2890	1684/3780	1509/3331	1147/2326	
Whey-containing dairy (150 g/day)	0.96 (0.86, 1.06)	1.09 (1.03, 1.16)	0.98 (0.93, 1.03)	1.00 (0.95, 1.06)	1.03 (0.96, 1.10)	0.90
Cereal fibre (10 g/day)	0.96 (0.70, 1.30)	0.84 (0.65, 1.09)	0.85 (0.68, 1.05)	0.96 (0.77, 1.21)	1.07 (0.84, 1.36)	0.79
Coffee (125 g/day)	0.99 (0.94, 1.04)	0.97 (0.93, 1.02)	0.97 (0.94, 1.00)	0.96 (0.92, 0.99)	0.95 (0.90, 0.99)	0.005
*n*_cases_/*n*_total_	489/1304	945/2338	1383/3131	1249/2776	972/1984	
Olive oil^b^ (10 g/day)	0.88 (0.75, 1.03)	0.98 (0.88, 1.08)	0.97 (0.88, 1.06)	0.96 (0.88, 1.05)	0.98 (0.89, 1.08)	0.27

Cox regression models were stratified by centre and age (years) and adjusted for sex, centre, educational attainment, physical activity, smoking status, BMI, alcohol consumption, soft drink consumption, fruit and vegetable intake, meat intake, total energy intake and mutual adjustment of the four dietary factors of interest (whey-containing dairy, cereal fibre, olive oil and coffee)

^a^The genetic risk score was constructed using the risk alleles for *TCF7L2* rs7903146, *KCNQ1* rs163184, *KCNQ1* rs2237892, *GIPR* rs10423928 and *WFS1* rs10010131

^b^Participants in the UK centres (*n*_cases_ = 685/*n*_total_ = 1594) and the Swedish centre Umeå (*n*_cases_ = 785/*n*_total_ = 1693) were excluded from specific analysis due to inaccurate assessment of olive oil consumption

Sensitivity analyses of the statistically significant interactions were carried out excluding participants with prevalent stroke, myocardial infarction and cancer as well as cases occurring within the first two years of follow-up to examine the possibility of confounding and reverse causation by these factors. No notable changes in effect estimates and *p* values were observed. Since olive oil consumption varied considerably between countries (ESM Table 2), we performed further sensitivity analysis specifically for all interactions involving olive oil. First, we excluded centres with extremely low consumption of olive oil (all centres where the median intake was 0), which included all of the centres in France, the Netherlands and Sweden. In this analysis, estimates and *p* values did not change notably. Second, we assessed interactions between each SNP and a binary variable indicating consumption vs non-consumption of olive oil. No interaction effects between olive oil consumption and any of the SNPs of interest were identified (data not shown).

## Discussion

### Summary of findings

Our primary aims were to investigate the presence of multiplicative interactions between the intake of foods as well as beverages and specific SNPs relevant to the incretin system, and to identify how these interactions affect incident type 2 diabetes in a European case-cohort study. We identified a possible interaction between *TCF7L2* and coffee, with carriers of the type 2 diabetes risk-conferring T allele benefiting more from coffee consumption than non-carriers. As far as the other genes are concerned, no compelling evidence of interaction was observed. The genetic risk score also showed evidence for an interaction with coffee. None of the aforementioned interactions passed the *p* value threshold for statistical significance after correction for multiple testing, thus all nominally significant findings should be interpreted with caution, as we cannot exclude the possibility of a Type I error. On the other hand, since very large sample sizes are required to detect gene–environment interactions of small magnitude, Type II errors are also possible.

### Gene–diet interactions involving *TCF7L2*

In line with the current findings, previous studies have also identified interactions between *TCF7L2* variants and dietary components [41–43], but none of these studies investigated an interaction with coffee. In our study, we found a nominally significant interaction between SNP rs12255372 in *TCF7L2* and coffee consumption, where the inverse association of coffee intake and type 2 diabetes was only present among participants carrying the risk-conferring T allele and was stronger in homozygous compared with heterozygous carriers.

The evidence from previous studies regarding interactions between *TCF7L2* variants and dietary components has focused mainly on wholegrain cereal and dietary fibre. Previous studies [41–43] reported that *TCF7L2* rs7903146 interacted with whole grains and cereal fibre intake, with the protective effect of dietary fibre appearing only in individuals homozygous for the C allele. In our study, we did find a similar trend of a protective association between cereal fibre and type 2 diabetes only among *TCF7L2* rs7903146 CC carriers and rs12255372 GG carriers, but these interactions did not reach statistical significance (*p* = 0.30 and *p* = 0.08, respectively). Similarly, a previous study also failed to show an interaction between *TCF7L2* rs12255372 and dietary fibre on type 2 diabetes risk [44]. Furthermore, another large study of 46,000 individuals failed to find an interaction between *TCF7L2* rs4506565 (a SNP in high LD with rs7903146) and intake of wholegrain cereal in relation to blood glucose and insulin levels [45]. Although these studies show a trend towards an interaction, even larger sample sizes or improved assessment methods may be needed to achieve the appropriate statistical power for detecting this specific interaction. We have to acknowledge, however, that overall no association between cereal fibre and risk for type 2 diabetes was detected in the EPIC-InterAct study, while such an association was observed in many other prospective cohort studies resulting in a clearly significant protective effect in a meta-analysis [28].

### Potential mechanisms

Our analyses show some evidence for a possible *TCF7L2*–coffee interaction, as well as an overall interaction of an incretin-specific genetic risk score with coffee on type 2 diabetes risk. The genetic risk score included SNPs preselected based on their involvement in the incretin system, therefore it may represent an overall genetic predisposition to defects in postprandial insulin secretion, which is somewhat attenuated by coffee consumption. TCF7L2 is a transcription factor for proteins involved in the proper functioning of the Wnt signalling pathway, essential for insulin secretion and beta cell proliferation. Being in positive feedback with GLP-1, TCF7L2 is believed to enhance pancreatic incretin sensitivity [13], as well as increase the production of GIP [14]. KCNQ1 plays an important role in the transport of incretins in enterocytes by exocytosis and thus incretin secretion from the gut [3, 10]. GIPR is the receptor for GIP and thus is directly involved in pancreatic incretin sensitivity [2, 3], while *WFS1* codes for Wolframin, which is responsible for the proper folding of proinsulin, and is thus linked to pancreatic incretin sensitivity [3, 9].

A study by Faerch et al [46] suggests that a major effect of *TCF7L2* variants on type 2 diabetes is mediated through lower secretion of GIP. In addition, the type 2 diabetes risk allele (T) in *TCF7L2* rs7903146 and rs12255372 has been linked to impaired insulin secretion and incretin action [7, 8], and more specifically, a reduction in the second phase of GLP-1-induced insulin secretion [8]. Given this, it would be rational to speculate that foods and beverages which tend to stimulate the secretion of incretins, such as coffee, will have the tendency to compensate, partly, for any genetic defects in the incretin system and thus confer higher protection from type 2 diabetes in individuals carrying the predisposing polymorphisms. Consistent with our results, a randomised crossover trial among individuals with type 2 diabetes that investigated the postprandial effects of three different isoenergetic diets on glucose levels concluded that a dietary pattern characterised by high coffee consumption resulted in a more pronounced insulin response with similar postprandial glucose levels; an effect attributed to increased GIP hormone release [47]. This effect of coffee is possibly brought about via delayed intestinal glucose absorption [24], but pathways involving direct effects on beta cells cannot be excluded; thus, further experimental studies that take incretin levels into account are required to elucidate the exact mechanisms.

### Study strengths and limitations

Study power is a major issue in gene–environment interaction studies. Many existing gene–environment interaction studies of type 2 diabetes are underpowered, particularly if the interactions are of small magnitude [48]. With a sample of approximately 20,000 our study is adequately powered for detecting gene–environment interactions of moderate magnitude. With 8291 cases of type 2 diabetes, our gene–environment interaction analysis is the largest ever reported from a single study. Still, we did not find an interaction that passed the multiple testing significance threshold, suggesting that either no interactions exist in the study population or that even larger sample sizes are required to detect them.

The fact that our study includes participants from eight different European countries significantly increases the external validity of our findings, at least in individuals of European origin.

Despite accurate assessment of both exposure (dietary factors) and outcome (diabetes) and the temporal relationship between the two, the inherent issue of dietary misreporting, which affects all observational studies, cannot be completely excluded.

The selection of dietary factors in our analysis was based on a review of the existing literature with the aim of identifying factors that have been shown to stimulate incretin release over and above macronutrient intake. However, we have to acknowledge that there are certainly more dietary factors and SNPs that influence the incretin system that are yet to be discovered. Hence, our analysis cannot be viewed as a comprehensive report on all possible interactions between SNPs and dietary factors in the incretin system.

## Conclusion

Our population-based case–cohort study provides evidence for the possible interaction of a *TCF7L2* variant and an incretin-specific genetic risk score with coffee consumption affecting the risk of type 2 diabetes. Further adequately powered studies or meta-analyses of smaller studies are required to replicate and confirm these interactions.

## Electronic supplementary material

Below is the link to the electronic supplementary material.

ESM 1 (PDF 71.0 kb)

ESM 2 (PDF 203 kb)

## Abbreviations

CEU: Utah Residents with Northern and Western European Ancestry
ENDB: EPIC nutrient database
EPIC: European Prospective Investigation into Cancer and Nutrition
GIP: Glucose-dependent insulinotropic peptide
GIPR: Gastric inhibitory polypeptide receptor
GLP-1: Glucagon-like peptide-1
GWAS: Genome-wide association studies
IQR: Interquartile range
KCNQ1: Potassium voltage-gated channel subfamily Q member 1
LD: Linkage disequilibrium
SNP: Single nucleotide polymorphism
TCF7L2: Transcription factor 7-like 2
WFS1: Wolframin ER transmembrane glycoprotein

## References

[1] Holst JJ, Vilsboll T, Deacon CF (2009). The incretin system and its role in type 2 diabetes mellitus. Mol Cell Endocrinol.

[2] Saxena R, Hivert MF, Langenberg C (2010). Genetic variation in GIPR influences the glucose and insulin responses to an oral glucose challenge. Nat Genet.

[3] Mussig K, Staiger H, Machicao F, Haring HU, Fritsche A (2010). Genetic variants affecting incretin sensitivity and incretin secretion. Diabetologia.

[4] Liu Z, Habener JF (2008). Glucagon-like peptide-1 activation of TCF7L2-dependent Wnt signaling enhances pancreatic beta cell proliferation. J Biol Chem.

[5] Loder MK, da Silva Xavier G, McDonald A, Rutter GA (2008). TCF7L2 controls insulin gene expression and insulin secretion in mature pancreatic beta-cells. Biochem Soc Trans.

[6] Shu L, Matveyenko AV, Kerr-Conte J, Cho JH, McIntosh CH, Maedler K (2009). Decreased TCF7L2 protein levels in type 2 diabetes mellitus correlate with downregulation of GIP- and GLP-1 receptors and impaired beta-cell function. Hum Mol Genet.

[7] Lyssenko V, Lupi R, Marchetti P (2007). Mechanisms by which common variants in the TCF7L2 gene increase risk of type 2 diabetes. J Clin Invest.

[8] Schäfer SA, Tschritter O, Machicao F (2007). Impaired glucagon-like peptide-1-induced insulin secretion in carriers of transcription factor 7-like 2 (TCF7L2) gene polymorphisms. Diabetologia.

[9] Schafer SA, Mussig K, Staiger H (2009). A common genetic variant in WFS1 determines impaired glucagon-like peptide-1-induced insulin secretion. Diabetologia.

[10] Yasuda K, Miyake K, Horikawa Y (2008). Variants in KCNQ1 are associated with susceptibility to type 2 diabetes mellitus. Nat Genet.

[11] Unoki H, Takahashi A, Kawaguchi T (2008). SNPs in KCNQ1 are associated with susceptibility to type 2 diabetes in East Asian and European populations. Nat Genet.

[12] Tan JT, Nurbaya S, Gardner D, Ye S, Tai ES, Ng DPK (2009). Genetic variation in KCNQ1 associates with fasting glucose and beta-cell function a study of 3,734 subjects comprising three ethnicities living in Singapore. Diabetes.

[13] Nilsson M, Holst JJ, Bjorck IME (2007). Metabolic effects of amino acid mixtures and whey protein in healthy subjects: studies using glucose-equivalent drinks. Am J Clin Nutr.

[14] Nilsson M, Stenberg M, Frid AH, Holst JJ, Bjorck IM (2004). Glycemia and insulinemia in healthy subjects after lactose equivalent meals of milk and other food proteins: the role of plasma amino acids and incretins. Am J Clin Nutr.

[15] Ma J, Stevens JE, Cukier K (2009). Effects of a protein preload on gastric emptying, glycemia, and gut hormones after a carbohydrate meal in diet-controlled type 2 diabetes. Diabetes Care.

[16] Hall WL, Millward DJ, Long SJ, Morgan LM (2003). Casein and whey exert different effects on plasma amino acid profiles, gastrointestinal hormone secretion and appetite. Br J Nutr.

[17] Tessari P, Kiwanuka E, Cristini M (2007). Slow versus fast proteins in the stimulation of beta-cell response and the activation of the entero-insular axis in type 2 diabetes. Diabetes Metab Res Rev.

[18] Holt SHA, Miller JCB, Petocz P (1997). An insulin index of foods: the insulin demand generated by 1000-kJ portions of common foods. Am J Clin Nutr.

[19] Thomsen C, Rasmussen O, Lousen T (1999). Differential effects of saturated and monounsaturated fatty acids on postprandial lipemia and incretin responses in healthy subjects. Am J Clin Nutr.

[20] Thomsen C, Storm H, Holst JJ, Hermansen K (2003). Differential effects of saturated and monounsaturated fats on postprandial lipemia and glucagon-like peptide 1 responses in patients with type 2 diabetes. Am J Clin Nutr.

[21] Gunnarsson PT, Winzell MS, Deacon CF (2006). Glucose-induced incretin hormone release and inactivation are differently modulated by oral fat and protein in mice. Endocrinology.

[22] Weickert MO, Mohlig M, Koebnick C (2005). Impact of cereal fibre on glucose-regulating factors. Diabetologia.

[23] Najjar AM, Parsons PM, Duncan AM, Robinson LE, Yada RY, Graham TE (2009). The acute impact of ingestion of breads of varying composition on blood glucose, insulin and incretins following first and second meals. Br J Nutr.

[24] Johnston KL, Clifford MN, Morgan LM (2003). Coffee acutely modifies gastrointestinal hormone secretion and glucose tolerance in humans: glycemic effects of chlorogenic acid and caffeine. Am J Clin Nutr.

[25] Ley SH, Hamdy O, Mohan V, Hu FB (2014). Prevention and management of type 2 diabetes: dietary components and nutritional strategies. Lancet.

[26] Sluijs I, Forouhi NG, Beulens JW (2012). The amount and type of dairy product intake and incident type 2 diabetes: results from the EPIC-InterAct Study. Am J Clin Nutr.

[27] Buijsse B, Boeing H, Drogan D (2015). Consumption of fatty foods and incident type 2 diabetes in populations from eight European countries. Eur J Clin Nutr.

[28] The InterAct Consortium (2015). Dietary fibre and incidence of type 2 diabetes in eight European countries: the EPIC-InterAct study and a meta-analysis of prospective studies. Diabetologia.

[29] The InterAct Consortium (2011). Design and cohort description of the InterAct project: an examination of the interaction of genetic and lifestyle factors on the incidence of type 2 diabetes in the EPIC study. Diabetologia.

[30] Slimani N, Deharveng G, Unwin I (2007). The EPIC nutrient database project (ENDB): a first attempt to standardize nutrient databases across the 10 European countries participating in the EPIC study. Eur J Clin Nutr.

[31] Cust AE, Skilton MR, van Bakel MM (2009). Total dietary carbohydrate, sugar, starch and fibre intakes in the European prospective investigation into cancer and nutrition. Eur J Clin Nutr.

[32] Riboli E, Hunt KJ, Slimani N (2002). European Prospective Investigation into Cancer and Nutrition (EPIC): study populations and data collection. Public Health Nutr.

[33] Wareham NJ, Jakes RW, Rennie KL (2003). Validity and repeatability of a simple index derived from the short physical activity questionnaire used in the European Prospective Investigation into Cancer and Nutrition (EPIC) study. Public Health Nutr.

[34] Haftenberger M, Lahmann PH, Panico S (2002). Overweight, obesity and fat distribution in 50- to 64-year-old participants in the European Prospective Investigation into Cancer and Nutrition (EPIC). Public Health Nutr.

[35] Langenberg C, Sharp SJ, Franks PW (2014). Gene-lifestyle interaction and type 2 diabetes: the EPIC interact case-cohort study. PLoS Med.

[36] Gaulton KJ, Ferreira T, Lee Y (2015). Genetic fine mapping and genomic annotation defines causal mechanisms at type 2 diabetes susceptibility loci. Nat Genet.

[37] Willett WC, Howe GR, Kushi LH (1997). Adjustment for total energy intake in epidemiologic studies. Am J Clin Nutr.

[38] Murphy N, Norat T, Ferrari P (2012). Dietary fibre intake and risks of cancers of the colon and rectum in the European prospective investigation into cancer and nutrition (EPIC). PLoS One.

[39] Buckland G, Mayen AL, Agudo A (2012). Olive oil intake and mortality within the Spanish population (EPIC-Spain). Am J Clin Nutr.

[40] Li J, Ji L (2005). Adjusting multiple testing in multilocus analyses using the eigenvalues of a correlation matrix. Heredity.

[41] Fisher E, Boeing H, Fritsche A, Doering F, Joost HG, Schulze MB (2009) Whole-grain consumption and transcription factor-7-like 2 (TCF7L2) rs7903146: gene-diet interaction in modulating type 2 diabetes risk. Br J Nutr 101:478–481 10.1017/S0007114508020369 19149908

[42] Hindy G, Sonestedt E, Ericson U (2012). Role of TCF7L2 risk variant and dietary fibre intake on incident type 2 diabetes. Diabetologia.

[43] Wirstrom T, Hilding A, Gu HF, Ostenson CG, Bjorklund A (2013). Consumption of whole grain reduces risk of deteriorating glucose tolerance, including progression to prediabetes. Am J Clin Nutr.

[44] Cornelis MC, Qi L, Kraft P, Hu FB (2009). TCF7L2, dietary carbohydrate, and risk of type 2 diabetes in US women. Am J Clin Nutr.

[45] Nettleton JA, McKeown NM, Kanoni S (2010). Interactions of dietary whole-grain intake with fasting glucose- and insulin-related genetic loci in individuals of European descent: a meta-analysis of 14 cohort studies. Diabetes Care.

[46] Faerch K, Pilgaard K, Knop FK (2013). Incretin and pancreatic hormone secretion in Caucasian non-diabetic carriers of the TCF7L2 rs7903146 risk T allele. Diabetes Obes Metab.

[47] Fernemark H, Jaredsson C, Bunjaku B, Rosenqvist U, Nystrom FH, Guldbrand H (2013). A randomized cross-over trial of the postprandial effects of three different diets in patients with type 2 diabetes. PLoS One.

[48] Franks PW, Pearson E, Florez JC (2013). Gene-environment and gene-treatment interactions in type 2 diabetes: progress, pitfalls, and prospects. Diabetes Care.

