# Supplementary material for: Investigation of gene–diet interactions in the incretin system and risk of type 2 diabetes: the EPIC-InterAct study
Source: Diabetologia. 2016 Sep 13;59(12):2613–21. doi: 10.1007/s00125-016-4090-5 (PMC6518069; doi:10.1007/s00125-016-4090-5)
Supplement: Supplementary file 1 — (PDF 71.0 kb) [file 125_2016_4090_MOESM1_ESM.pdf]

The InterAct Consortium list of authors is as follows:

Alexandros Heraclides† (Department of Molecular Epidemiology, German Institute of Human Nutrition Potsdam-Rehbruecke, Nuthetal, Germany and Centre for Primary Care and Population Health, Medical School, University of Nicosia, Nicosia, Cyprus), **Karina Meidtner†** (Department of Molecular Epidemiology, German Institute of Human Nutrition Potsdam-Rehbruecke, Nuthetal, Germany and German Center for Diabetes Research (DZD), Neuherberg, Germany), **Brian Buijsse** (Department of Epidemiology, German Institute of Human Nutrition Potsdam-Rehbruecke, Nuthetal, Germany), **Yvonne T. van der Schouw** (Department of Epidemiology, Julius Center for Health Sciences and Primary Care University Medical Center Utrecht, Utrecht, The Netherlands), **Ivonne Sluijs** (Department of Epidemiology, Julius Center for Health Sciences and Primary Care University Medical Center Utrecht, Utrecht, The Netherlands), **Daphne L van der A** (National Institute for Public Health and the Environment, Bilthoven, The Netherlands), **Anneleen Kuijsten** (Division of Human Nutrition, Wageningen University, Wageningen, The Netherlands), **Antonio Agudo** (Unit of Nutrition and Cancer, Cancer Epidemiology Research Programme, Catalan Institute of Oncology (ICO), Barcelona, Spain), **Eva Ardanaz** (Navarra Public Health Institute, Pamplona, Spain and IdiSNA, Navarra Institute for Health Research, Pamplona, Spain and CIBER Epidemiology and Public Health CIBERESP, Madrid, Spain), **Heiner Boeing** (Department of Epidemiology, German Institute of Human Nutrition Potsdam-Rehbruecke, Nuthetal, Germany), **Edith JM Feskens** (Division of Human Nutrition, Wageningen University, Wageningen, The Netherlands), **Diana Gavrila** (Department of Epidemiology, Murcia Regional Health Council, Murcia, Spain and CIBER Epidemiología y Salud Pública (CIBERESP), Madrid, Spain), **Verena Katzke** (Division of Cancer Epidemiology, German Cancer Research Center [DKFZ], Heidelberg, Germany), **Timothy J. Key** (Cancer Epidemiology Unit, Nuffield Department of Population Health, University of Oxford, Oxford, UK), **Tilman Kühn** (Division of Cancer Epidemiology, German Cancer Research Center [DKFZ], Heidelberg, Germany), **Vittorio Krogh** (Nutritional Epidemiology Unit, National Cancer Institute, Milan, Italy), **Cecilie Kyrø** (Danish Cancer Society Research Center, Copenhagen, Denmark), **Elena Molina-Portillo** (Granada Cancer Registry, Andalusian School of Public Health, Granada, Spain), **Lotte Maxild Mortensen** (Department of Cardiology, Aalborg University Hospital, Aalborg, Denmark and Department of Public Health, Section for Epidemiology, Aarhus University, Aarhus, Denmark), **Peter M. Nilsson** (Department of Clinical Sciences, Lund University, Malmö, Sweden), **Kim Overvad** (Department of Public Health, Section for Epidemiology, Aarhus University, Denmark), **Domenico Palli** (Molecular and Nutritional Epidemiology Unit, Cancer Research and Prevention Institute – ISPO, Florence, Italy), **Salvatore Panico** (Dipartimento de Medicina Clinica e Chirurgia, Federico II University, Naples, Italy), **Fulvio Ricceri** (Unit of Epidemiology, Regional Health Service ASL TO3,Grugliasco, Italy and Unit of Cancer Epidemiology, Department of Medical Sciences, University of Turin, Turin, Italy), **Rosario Tumino**

(Cancer Registry and Histopathology Unit, 'Civic – M.P.Arezzo' Hospital, Ragusa, Italy), **Nita G. Forouhi** (MRC Epidemiology Unit, University of Cambridge, School of Clinical Medicine, Institute of Metabolic Science, Cambridge Biomedical Campus, Cambridge, UK)**, Stephen J Sharp** (MRC Epidemiology Unit, University of Cambridge, School of Clinical Medicine, Institute of Metabolic Science, Cambridge Biomedical Campus, Cambridge, UK), **Claudia Langenberg** (MRC Epidemiology Unit, University of Cambridge, School of Clinical Medicine, Institute of Metabolic Science, Cambridge Biomedical Campus, Cambridge, UK)**, Robert Scott** (MRC Epidemiology Unit, University of Cambridge, Cambridge, United Kingdom)**, Paul W. Franks** (Department of Public Health and Clinical Medicine, Umeå University, Umeå, Sweden, and Department of Clinical Sciences, Lund University, Malmö, Sweden)**, Matthias B. Schulze** (Department of Molecular Epidemiology, German Institute of Human Nutrition Potsdam-Rehbruecke, Nuthetal, Germany and German Center for Diabetes Research (DZD), Neuherberg, Germany), **Elio Riboli** (Department of Epidemiology and Biostatistics, School of Public Health, Imperial College London, London, UK), **Nicholas J. Wareham** (MRC Epidemiology Unit, University of Cambridge, School of Clinical Medicine, Institute of Metabolic Science, Cambridge Biomedical Campus, Cambridge, UK)

†authors contributed equally to this work
