# Supplementary material for: Investigation of gene–diet interactions in the incretin system and risk of type 2 diabetes: the EPIC-InterAct study
Source: Diabetologia. 2016 Sep 13;59(12):2613–21. doi: 10.1007/s00125-016-4090-5 (PMC6518069; doi:10.1007/s00125-016-4090-5)
Supplement: Supplementary file 2 — (PDF 203 kb) [file 125_2016_4090_MOESM2_ESM.pdf]

ESM Table 1: Characteristics of the incretin-related SNPs used for the current analysis in the EPIC-InterAct study

SNP	Gene	Chromosome	Alleles (major/minor)	MAF[a]	Risk allele	Risk allele possible effect
rs12255372	*TCF7L2*	10	G/T	0.30	T	decreased pancreatic GLP-1 sensitivity and lower GIP secretion [4-8, 14]
rs7903146	*TCF7L2*	10	C/T	0.30	T	
rs163171	*KCNQ1*	11	C/T	0.22	T	decreased GLP-1 and GIP secretion from the gut [10-12]
rs163184	*KCNQ1*	11	T/G	0.48	G	
rs2237892	*KCNQ1*	11	C/T	0.06	C	
rs10423928	*GIPR*	19	T/A	0.20	A	decreased pancreatic GIP sensitivity [2]
rs10010131	*WFS1*	4	G/A	0.40	G	decreased pancreatic GLP-1 sensitivity [9]

[a]Minor allele frequency

ESM Table 2: Baseline characteristics of the EPIC-InterAct subcohort participants with data available for at least one SNP of interest stratified by country

	France (N=311)			Italy (N=1539)			Spain (N=2657)			UK (N=933)		
Characteristic	Missing Data (%)	Median / %	25th - 75th Percentile / N	Missing Data (%)	Median / %	25th - 75th Percentile / N	Missing Data (%)	Median / %	25th - 75th Percentile / N	Missing Data (%)	Median / %	25th - 75th Percentile / N
Age (years)	0	55.2	(50.8 - 61.1)	0	50.1	(44.0 - 56.5)	0	48.2	(42.6 - 54.9)	0	56.8	(48.8 - 65.4)
Sex (female)	0	100.0	311	0	65.4	1007	0	62.6	1663	0	61.0	569
BMI (kg/m^2)	0.3	22.4	(20.7 - 24.4)	0	25.2	(22.8 - 27.9)	0	27.8	(25.3 - 30.5)	0	24.9	(22.8 - 27.4)
Waist (cm)	0	74.0	(70.0 - 80.0)	1.9	83.0	(74.0 - 91.0)	0.1	92.5	(83.0 - 100.0)	0.8	83.0	(73.6 - 91.0)
Highest School Level	0			0			0			0		
None		0.6	2		1.0	16		33.9	901		0.0	0
Primary		11.6	36		47.1	725		39.1	1040		31.7	296
Technical		0			12.5	192		7.6	202		35.2	328
Secondary		51.8	161		25.0	385		6.9	183		10.9	102
Further education		36.0	112		14.4	221		12.5	331		22.2	207
Smoking status	0			0			0			0		
Never		70.4	219		44.7	688		54.5	1447		51.0	476
Former		20.9	65		27.6	424		17.0	451		34.4	321
Current		8.7	27		27.8	427		28.6	759		14.6	136
Cambridge Index of Physical Activity	0			0			0			0		
Inactive		18.3	57		26.3	404		37.1	986		31.9	298
Moderately inactive		34.4	107		39.9	614		34.3	912		31.5	294
Moderately active		37.9	118		17.9	275		17.1	453		21.7	202
Active		9.3	29		16.0	246		11.5	306		14.9	139
Energy (MJ/day)	0	8.9	(7.5 – 10.7)	0	9.4	(7.5 – 11.4)	0	8.7	(7.0 – 10.8)	0	8.2	(6.6 – 10.0)
Cereal fibre (g/day)	0.3	5.7	(3.9 - 7.7)	0	7.1	(5.1 - 9.8)	0	5.6	(3.7 - 7.8)	0	6.5	(4.3 - 9.9)
Fruit and vegetables (g/day)	0	417	(293 - 567)	0	424	(306 - 565)	0	466	(310 - 651)	0	305	(211 - 435)
Whey-containing dairy products (g/day)	0	205	(121 - 323)	0	141	(27 - 232)	0	238	(150 - 357)	0	373	(293 - 518)
Meat (g/day)	0	77.6	(47.8 – 112.4)	0	67.3	(44.3 – 96.7)	0	71.0	(43.3 – 107.5)	0	55.1	(32.1 – 87.5)
Olive oil (g/day)	0	0.0	(0.0 - 2.4)	0	23.2	(16.9 - 32.0)	0	19.7	(10.3 – 29.9)	100		
Coffee (g/day)	0	250	(70 - 415)	0	90	(60 - 130)	0	89	(11 - 173)	0	475	(83 - 488)
Soft drinks (g/day)	0	0.0	(0.0 - 0.0)	0	0.0	(0.0 - 28.6)	0	0.0	(0.0 - 0.0)	0	33.1	(0.0 - 158.0)
alcohol intake (g/day)	0			0			0			0		
0		16.4	51		15.5	239		33.6	893		14.5	135
>0-6		36.7	114		32.8	504		24.1	639		42.7	398
>6-12		14.8	46		10.7	164		9.6	256		20.5	191
>12-24		20.6	64		16.1	248		12.2	325		14.3	133
>24		11.6	36		25.0	384		20.5	544		8.2	76
Hypertension	0.3	13.5	42	0.2	19.2	296	<0.1	17.80	474	0.3	12.8	119
Hyperlipidaemia	1.3	11.3	35	0.2	23.9	368	0.7	18.7	498	0.8	8.0	75
Stroke	1.9	0.3	1	0.3	0.2	3	0.2	0.4	11	0.4	0.8	7
Myocardial infarction	0	0.3	1	0	0.5	7	<0.1	0.4	11	0.9	2.6	24

ESM Table 2 (continued): Baseline characteristics of the EPIC-InterAct subcohort participants with data available for at least one SNP of interest stratified by country

	Netherlands (N=1171)			Germany (N=1819)			Sweden (N=2605)		
Characteristic	Missing Data (%)	Median / %	25th - 75th Percentile / N	Missing Data (%)	Median / %	25th - 75th Percentile / N	Missing Data (%)	Median / %	25th - 75th Percentile / N
Age (years)	0	53.4	(49.5 – 60.0)	0	50.4	(43.0 - 57.6)	0	53.6	(48.5 - 60.7)
Sex (female)	0	82.8	970	0	58.7	1068	0	56.7	1477
BMI (kg/m^2)	0	24.7	(22.7 - 27.4)	0	25.2	(22.9 - 27.9)	0	24.6	(22.4 - 27.2)
Waist (cm)	0	82.0	(75.0 - 89.0)	0	85.0	(75.6 - 94.0)	36.2	83.0	(74.0 - 93.0)
Highest School Level	0			0			0		
None		0.0	0		0.7	13		0.4	11
Primary		18.1	212		19.9	362		36.2	942
Technical		30.0	351		37.9	690		25.1	655
Secondary		29.7	348		6.5	118		17.2	448
Further education		22.2	260		35.0	636		21.1	549
Smoking status	0			0			0		
Never		40.1	469		47.2	858		46.0	1199
Former		32.5	380		32.4	589		27.3	711
Current		27.5	322		20.5	372		26.7	695
Cambridge Index of Physical Activity	0			0			0		
Inactive		7.4	87		15.8	288		24.3	634
Moderately inactive		27.6	323		37.5	682		33.3	868
Moderately active		24.9	292		26.9	490		25.7	670
Active		40.1	469		19.7	359		16.6	433
Energy (MJ/day)	0	7.9	(6.6 – 9.4)	0	8.1	(6.7 – 10.1)	0	8.5	(6.9 – 10.4)
Cereal fibre (g/day)	0	7.2	(5.4 - 9.6)	0	9.1	(6.4 - 12.4)	0	8.4	(5.9 - 11.9)
Fruit and vegetables (g/day)	0	278	(188 - 388)	0	191	(144 - 273)	0	240	(146 - 360)
Whey-containing dairy products (g/day)	0	365	(213 - 541)	0	164	(84 - 281)	0	330	(197 - 512)
Meat (g/day)	0	79.3	(49.5 – 111.8)	0	82.0	(52.1 – 119.9)	0	64.0	(41.8 – 93.8)
Olive oil (g/day)	0	0.0	(0.0 - 0.0)	0	0.6	(0.0 - 2.0)	36.2	0.0	(0.0 - 0.0)
Coffee (g/day)	0	500	(375 - 750)	0	392	(261 - 580)	0	400	(300 - 601)
Soft drinks (g/day)	0	55.7	(9.2 - 138.5)	0	2.0	(0.0 - 29.3)	0	39.4	(0.0 - 126.9)
alcohol intake (g/day)	0			0			0		
0		15.9	186		4.00	72		13.5	352
>0-6		38.5	451		36.4	662		46.4	1208
>6-12		14.5	170		18.0	328		19.0	494
>12-24		16.7	196		19.6	357		14.8	386
>24		14.4	168		22.0	400		6.3	165
Hypertension	4.1	18.3	214	0.2	31.0	563	0.2	15.7	408
Hyperlipidaemia	8.3	8.5	99	0.5	29.2	531	99.6	0.4	11
Stroke	0.5	0.9	11	0	1.0	19	43.2	1.0	26
Myocardial infarction	0.0	1.6	19	0	1.5	27	7.4	1.7	45

ESM Table 3: Hazard Ratios (95% CI) for incident type 2 diabetes by genotype of each SNP, as well as the genetic risk score, in the EPIC-InterAct study

**SNP**	**HR (95% CI)** major allele homozygous (n_{cases}/n_{total})	heterozygous (n_{cases}/n_{total})	minor allele homozygous (n_{cases}/n_{total})	***p-value (additive model)***
TCF7L2 rs12255372	GG (3,216/8,171)	GT (3,520/7,661)	TT (906/1,807)	
	1.00	1.40 (1.23; 1.61)	1.61 (1.37; 1.88)	$1.4*10^{-8}$
TCF7L2 rs7903146	CC (3,197/8,196)	CT (3,622/7,896)	TT (977/1,879)	
	1.00	1.39 (1.21; 1.60)	1.74 (1.51; 1.99)	$2.9*10^{-11}$
KCNQ1 rs163171	CC (4,647/10,652)	CT (2,605/6,110)	TT (391/883)	
	1.00	0.96 (0.87; 1.06)	1.04 (0.88; 1.23)	*0.74*
KCNQ1 rs163184	TT (2,007/4,850)	TG (3,971/9,135)	GG (1,977/4,367)	
	1.00	1.11 (1.03; 1.19)	1.19 (1.09; 1.30)	$6.2*10^{-5}$
KCNQ1 rs2237892	CC (6,148/13869)	CT (701/1,647)	TT (20/61)	
	1.00	0.90 (0.81; 1.00)	0.58 (0.30; 1.12)	*0.008*
GIPR rs10423928	TT (4,925/11,548)	TA (2,684/6,014)	AA (345/784)	
	1.00	1.10 (1.03; 1.17)	1.07 (0.92; 1.24)	*0.01*
WFS1 rs10010131	GG (2,857/6,392)	GA (3,424/8,095)	AA (1,185/2,773)	
	1.00	0.90 (0.83; 0.97)	0.89 (0.78; 1.01)	*0.02*

N (risk alleles)	0-3	4	5	6	7-10	continuous model	*p for trend*
n_{cases} / n_{total}	642/1,710	1,188/2,890	1,684/3,780	1,509/3,331	1,147/2,326	6,170/14,037	
Genetic risk score[a]	1.00	1.17 (0.97; 1.42)	1.42 (1.25;1.60)	1.49 (1.29; 1.73)	1.72 (1.50; 1.96)	1.17 (1.14; 1.22)	$2.4*10^{-20}$

Cox-regression stratified by centre and age (years) and adjusted for sex. Country-specific estimates were combined by random-effects meta-analysis.

[a] The genetic risk score was constructed using the type 2 diabetes risk allele for *TCF7L2 rs7903146*, *KCNQ1 rs163184*, *KCNQ1 rs2237892*, *GIPR rs10423928*, *WFS1 rs10010131*. For the continuous analysis the risk score was z-transformed (mean = 0, sd = 1).

ESM Table 4: Multivariable-adjusted Hazard Ratios (95% CI) for the dietary main effect on incident type 2 diabetes in the EPIC-InterAct study

**Dietary factor**	**HR (95% CI)**
n_{cases} / n_{total}	**8,086/18,638**
whey-containing dairy (per 150g/d)	1.02 (0.98; 1.06)
cereal fibre (per 10 g/d)	1.06 (0.93; 1.20)
Coffee (per 125 g/day)	0.98 (0.96; 0.99)
n_{cases} / n_{total}[a]	**6,616/15,351**
olive oil[a] (per 10 g/d)	0.97 (0.90; 1.03)

Analysis stratified by centre and age (years) and adjusted for sex, centre, educational attainment, physical activity, smoking status, BMI, alcohol consumption, soft drink consumption, fruit and vegetable intake, meat intake, total energy intake and mutual adjustment of the four dietary factors of interest (whey-containing-dairy, cereal fibre, olive oil and coffee)

[a] Participants in the UK centres (n_{cases} = 685 /n_{total} = 1,594)and the Swedish centre Umeå (n_{cases} = 785 / n_{total} = 1,693) were excluded from specific analysis due to inaccurate assessment of olive oil consumption.
